# Hydroxycinnamic Acids and Their Derivatives in *Broa*, a Traditional Ethnic Maize Bread

**DOI:** 10.3390/foods9101471

**Published:** 2020-10-15

**Authors:** Andreia Bento-Silva, Noélia Duarte, Elsa Mecha, Maria Belo, Maria Carlota Vaz Patto, Maria do Rosário Bronze

**Affiliations:** 1 FCT NOVA, Faculdade de Ciências e Tecnologia, Campus da Caparica, Universidade Nova de Lisboa, 2829-516 Caparica, Portugal; abentosilva@ff.ulisboa.pt; 2 ITQB NOVA, Instituto de Tecnologia Química e Biológica António Xavier, Universidade Nova de Lisboa, Avenida da República, Quinta do Marquês, 2780-157 Oeiras, Portugal; emecha@itqb.unl.pt (E.M.); mariabelo87@gmail.com (M.B.); cpatto@itqb.unl.pt (M.C.V.P.); 3 FFULisboa, Faculdade de Farmácia da Universidade de Lisboa, Avenida Professor Gama Pinto, 1649-003 Lisboa, Portugal; 4 iMed.ULisboa, Faculdade de Farmácia, Research Institute for Medicines, Universidade de Lisboa, Avenida Professor Gama Pinto, 1649-003 Lisboa, Portugal; mduarte@ff.ulisboa.pt; 5 iBET, Instituto de Biologia Experimental e Tecnológica, Avenida da República, Quinta do Marquês, 2780-157 Oeiras, Portugal

**Keywords:** maize, *broa*, hydroxycinnamic acid amides, ferulic acid, *p*-coumaric acid, dehydrodiferuloyl putrescines, dehydrotriferuloyl putrescines

## Abstract

Maize is one of the most interesting dietary sources of hydroxycinnamic acids, widely known for their beneficial health effects, namely antioxidant properties. This work aims to identify hydroxycinnamic acids and their derivatives in *broa*, a Portuguese traditional ethnic maize bread, and corresponding maize flours. Soluble and insoluble phenolic fractions of diverse maize flours and corresponding *broas* were prepared and analysed by HPLC-DAD-MS/MS (high-performance liquid chromatography coupled with diode array detector and tandem mass spectrometry). Besides free hydroxycinnamic acids, mainly ferulic and *p*-coumaric acids, several structural isomers and stereoisomers of insoluble ferulic acid dehydrodimers (n = 18) and trimers (n = 11), were also identified. Hydroxycinnamic acid amides consisting of coumaroyl and feruloyl conjugates (n = 22) were present in both soluble and insoluble fractions of maize flours and breads, in different isomeric forms. A new compound was putatively identified as *bis*-*N,N′*-diferuloyl putrescine. Additionally, more complex and insoluble hydroxycinnamic acid amides, derived from ferulic acid dehydrodimers (n = 47) and trimers (n = 18), were also putatively identified for the first time, suggesting that hydroxycinnamic acid amides are also linked to maize cell walls. Since hydroxycinnamic derivatives were not only identified in maize flours, but also in *broas*, they can contribute to the antioxidant properties and beneficial health effects of maize-based foods.

## 1. Introduction

Maize (*Zea mays*) is widely grown throughout the world, being considered a staple cereal in many countries, where it can be used to produce different food products [1]. In Portugal, maize is used to produce *broa*, a traditional ethnic bread prepared with whole grain maize (50–100%), and rye and/or wheat (0–50%) flours [2]. *Broa* was considered a hearty peasant bread and one of the 50 world’s best breads by CNN Travel, in October 2019 [3].

Among the phytochemicals present in cereals, phenolic compounds, namely hydroxycinnamic acids, contribute positively for human health [4,5] due to their antibacterial, anti-ageing, anti-carcinogenic, neuroprotective, cardiovascular and anti-diabetic properties [4]. In comparison to other cereals, whole maize grains contain higher levels of hydroxycinnamic acids [4].

Generally, phenolic compounds can be found in their soluble or insoluble forms. Soluble forms, also known as “extractable polyphenols” [6], can be present in their free form or conjugated with smaller molecules, such as simple sugars and amines [5,7,8,9]. Particularly abundant free hydroxycinnamic acids present in maize grains are ferulic (FA) and *p*-coumaric (pCA) acids. These acids can be conjugated with polyamines, yielding hydroxycinnamic acid amides (HCAAs), such as *N,N′*-feruloyl putrescine (DFP) [7,8,9].

The role of HCAAs in maize grains is not known for sure [5]. They may be associated with several processes, such as plant growth and development, floral induction and reproduction, cell division, control of intracellular polyamine concentrations, cell wall reinforcement, and plant adaptation to stress, such as resistance to cold or pathogen attack [9,10,11,12]. To the best of our knowledge, HCAAs have not been detected in maize-based foods.

The “insoluble phenolic compounds”, also known as “non-extractable polyphenols”, are mainly high molecular weight compounds mostly (>94%) bound to arabinoxylans [5,6]. They include, among others, dehydrodiferulic (DFAs), dehydrotriferulic (TFAs) and dehydrotetraferulic (TeFAs) acids [4,5]. These compounds are responsible for the cross-linking of cell wall polysaccharides, which is implicated in different processes in plants, such as the control of cellular expansion associated with growth [4]. Similar to HCAAs, cross-linking has been suggested to protect against pathogens, since its increase results in a thinner and firmer cell walls, therefore decreasing pathogen penetration into plant tissues [4].

It has been reported that HCAAs exert interesting antioxidant, anti-inflammatory, and chemopreventive properties [8,9,13,14,15,16]. Similarly, most DFAs have shown higher radical-scavenging efficacies than free FA [17]. Despite their possible benefits for human health, the presence of HCAAs, DFAs, TFAs, and TeFAs in maize-based foods has been poorly studied. The bioavailability of hydroxycinnamic acids depends on their presence in food matrices which is in turn affected by food processing [18]. In particular, the majority of bound compounds, such as DFAs, reaches the colon and need to be liberated from the food matrix by the action of enzymes during small intestinal digestion or colonic fermentation in order to be absorbed [18,19]. Conversely, soluble compounds are generally readily available for absorption [4].

In order to evaluate the importance and possible health effects associated with the consumption of maize products, hydroxycinnamic acids and their derivatives should be characterized and their bioaccessibility evaluated. The present study aimed at identifying the main phenolic compounds present in whole grain maize flours and *broas*, focusing on the identification of FA dimers and trimers, as well as hydroxycinnamic acid derivatives, including new insoluble dehydrodiferulic and dehydrotriferulic acid putrescines.

## 2. Materials and Methods

### 2.1. Maize Flour and Broas Preparation

Five traditional Portuguese open-pollinated maize varieties and a commercial maize flour (Nacional Type 175) were studied. Maize flours from the traditional varieties were obtained after milling the whole grain in an artisan water-mill with millstones (Moinhos do Inferno, Viseu, Portugal, Falling Number 3100). The commercial flour was acquired already milled. All flour samples are described in Table 1.

*Broas* (n = 6) were prepared in a bakery following a traditional recipe [21] and using the flours described in Table 1. The recipe included 70% maize flour, 20% commercial rye flour (Concordia type 70, Portugal) and 10% commercial wheat flour (National type 65, Portugal). A grinding mill (IKA MF 10.2, Königswinter, Germany) with a 1.5 mm sieve was used for milling *broas* just before the extraction procedure.

The commercial rye and wheat flours used in *broas* recipe were also studied.

### 2.2. Reagents

Absolute ethanol (EtOH), sodium hydroxide (NaOH), formic acid ≥ 95%, and all the standards used in the present work (ferulic, *p*-coumaric, *o*-coumaric, *m*-coumaric, *p*-hydroxybenzoic, caffeic, syringic, citric, vanillic, and protocatechuic acids, syringaldehyde, vanillin, quercetin, and kaempferol) were obtained from Sigma-Aldrich, St. Louis, MO, USA. Acetonitrile HPLC Plus Gradient grade, hexane, and ethyl acetate (EtOAc) were from Carlo Erba, Val de Reuil, France. Phosphoric acid 85% *p.a.* was from Panreac, Barcelona, Spain. Water was purified by a Milli-Q water purification system from Millipore, Burlington, MA, USA.

### 2.3. Extraction of Phenolic Compounds

**Figure 1:**
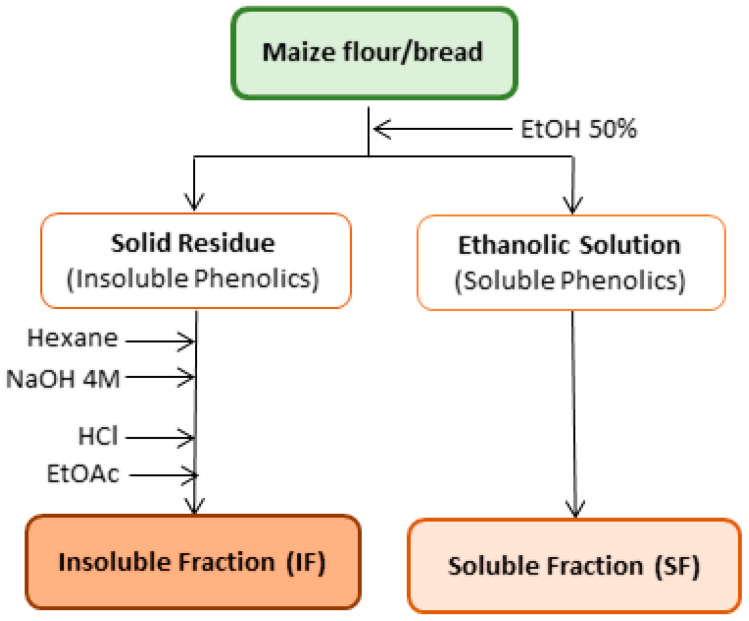
Representative scheme of the extraction procedure of the soluble and insoluble phenolic fractions.

A conventional extraction procedure [22] for raw maize, wheat, and rye flours and *broas* (4 g) was performed. Briefly, 4 g of maize flour was extracted with 20 mL of EtOH/H_2_O (50%, *v*/*v*) for 15 min, using an Ultra Turrax T25 (Janke & Kunkel, IKA Labortechnik, Burlington, Germany), at room temperature, yielding an ethanolic solution that contained the soluble phenolic compounds (SF) and a solid residue comprising the insoluble compounds (IF), as described in Figure 1.

In order to obtain the insoluble fraction (IF), the solid residue was defatted with hexane (3 × 20 mL), centrifuged (7000× *g*, 10 min), and hydrolysed with NaOH 4 M (60 mL, pH 14 ± 0.5), for 15 h at room temperature, in the presence of N_2_ [23,24]. After hydrolysis, the pH was set to 1.5 ± 0.5 with concentrated HCl and the solution was extracted with EtOAc (3 × 30 mL), evaporated until dryness through a SpeedVac (Labconco, Kansas City, MO, USA) and reconstituted in 20 mL of EtOH 50%. Both fractions (SF and IF) from maize flours and *broas* were prepared in duplicate, filtered through 0.20 μm polytetrafluoroethylen (PTFE) syringe filters (Chromafil^®^ Macherey-Nagel, Düren, Germany) and analysed by HPLC-DAD (high-performance liquid chromatography coupled with diode array detector). Extracts (10 mL) obtained from commercial rye and wheat flours and Verdeal de Aperrela maize flour and *broa* were concentrated until dryness and reconstituted in 500 µL of EtOH/H_2_O (50%, *v*/*v*) before HPLC-DAD-MS/MS (HPLC coupled with DAD and tandem mass spectrometry) analysis.

### 2.4. Analysis of Phenolic Compounds by Liquid Chromatography

In order to compare the phenolic composition, extracts from all samples, maize flour and corresponding *broa,* were analysed in a Thermo Fisher Scientific Surveyor HPLC system, equipped with a DAD (Waltham, MA, USA). The analytical conditions are described in Appendix A.

Extracts of the SF and IF of Verdeal de Aperrela sample, as well as the commercial wheat and rye flours, were analysed on an Alliance 2695 separation module HPLC system (Waters, Dublin, Ireland) coupled to a 2996 Photodiode Array Detector and a Micromass^®^ Quattro Micro triple quadrupole (TQ) (Waters, Dublin, Ireland). The analytical conditions are described in Appendix B. MS/MS experiments were performed in order to identify the major phenolic compounds. Additionally, when standards were commercially available, MS/MS conditions were optimised (Table S1) and extracts were analysed in multiple reaction monitoring (MRM) mode in order to increase selectivity and sensitivity.

In both types of equipment, the injection volume was 20 µL, and the chromatographic separation procedure was carried out using a Lichrocart^®^ RP-18 column (250 × 4 mm, 5 µm) and a Manu-cart^®^ RP-18 pre-column (Merck, Darmstadt, Germany) in a thermostated oven at 35 °C.

### 2.5. Data Analysis

ChromQuest (Thermo Fisher Scientific, Waltham, MA, USA) and MassLynx (Waters, Dublin, Ireland) software were used to control analytical conditions and collect data from HPLC-DAD and HPLC-DAD-MS/MS, respectively. For compounds identification purposes, mass and UV spectra were compared with spectra already published in the literature. When standards were commercially available, the identification was based on the comparison of their fragmentation patterns and retention times.

## 3. Results and Discussion

After a preliminary analysis by HPLC-DAD at 280 and 320 nm (maximum absorption of phenolic compounds and hydroxycinnamic acids) [24] it was possible to conclude that SF and IF fractions from all maize flours showed identical chromatographic profiles, but with differences in peaks’ intensity (Figure S1). Similar results were obtained from the comparison of *broas* (Figure S1). Therefore, aiming at characterising their phenolic composition, both fractions of a randomly chosen maize flour (Verdeal de Aperrela) and corresponding bread were analysed by HPLC-DAD-MS/MS. Rye and wheat flours used for *broa* production were also analysed. The putatively identified compounds are described in Table 2.

### 3.1. Small Phenolic Compounds

The main free phenolic compounds detected in both SF and IF of all raw flours (maize, rye, wheat) and *broas* were the *trans* isomeric forms of FA (16) and pCA (13) (Table 2). Their presence was confirmed by the comparison with the chromatograms and UV spectra of the respective commercial standards.

An additional peak at *m/z* 193 [M − H]^−^ was identified as *cis*-FA (17), especially evident in the IF of maize flours and *broas*. Previously, Guo et al. [34] identified isoferulic acid (*m/z* 193 [M − H]^−^) as one of the major components of cereal alkaline extracts, due to its unique fragmentation behaviour observed by HPLC-DAD-MS/MS analysis. However, in the present work, isoferulic acid was not detected in any of the samples analysed, since the retention time, UV and MS/MS spectra were not coincident with the reported data. Similarly, *cis*-pCA (15) was also identified in the SF and IF of all samples (raw flours and breads) studied.

Vanillic (6) and syringic (9) acids were also identified by commercial standards in the SF of all raw flours (Verdeal de Aperrela and commercial wheat and rye) and *broa*. Caffeic acid (8) was detected in the IF but not in the SF of all raw flours, suggesting it was linked to insoluble cereal components. Conversely, it was detected in the SF of *broas*, possibly due to its release from cellular vacuoles during processing, as previously suggested [35]. Other phenolic acids, such as *p*-hydroxybenzoic, *m*-, and *o*-coumaric acids, as well as some flavonoids, such as quercetin and kaempferol, have been reported in maize grains in low or trace amounts [5,29,36]. However, these compounds were not detected in any of the analysed samples. Protocatechuic acid (3) was detected in the IF of all raw flours and *broa*, but as caffeic acid, it was not extracted using the conventional ethanolic extraction procedure (SF), suggesting it was linked to insoluble cereals components. On the other hand, it was not detected in the SF of *broa*, meaning that it was degraded or not released during its processing.

Furthermore, it was possible to identify *p*-hydroxybenzaldehyde (10) and vanillin (11) in the SF and IF of all raw flours and *broa*. Syringaldehyde (14) was also detected in the SF of *broa*, maize, and rye, and in the IF of all samples. It has been described that vanillin and *p*-hydroxybenzaldehyde can be produced from FA and pCA, respectively [37].

Coumaroyl glycerol (12) was detected in the SF of maize and rye flours and *broa*, but not detected in the IF of any sample, meaning that, if present, it was possibly hydrolysed to free pCA during the IF extraction procedure.

Three FA hexosides (peaks 4, 5, 7: FA hexoside 1, 2, 3) were also detected in the SF of *broa*. In addition, FA hexoside 3 was also detected in the SF of wheat flour. A FA hexoside has been previously identified in wheat [26]. The detection of FA hexosides 1 and 2 in *broa* SF suggests that they should be present in at least one of the raw flours used for *broa* production (maize, wheat, or rye). However, since they were not detected in the SF of any raw flours, they were probably associated with insoluble compounds and hydrolysed to FA during the IF extraction procedure.

It is widely known that soluble phenolic acids, such as free FA and some small FA oligosaccharides, are readily available for absorption by the human gastrointestinal (GI) tract. Results obtained confirm that several small phenolic acids can be found in *broa* SF, being bioaccessible and able to reach the specific sites where they can exert their biological actions [4].

### 3.2. Ferulic Acid Dehydrodimers

In cereals, FA dimerises mainly by free phenoxyl radicals coupling reactions, at their O-4, C-5 or C-8 positions, yielding diferulate esters connected via 8-5′, 8-O-4′, 5-5′, 8-8′, and 4-O-5′ linkages (Figure S2).

**Figure 2:**
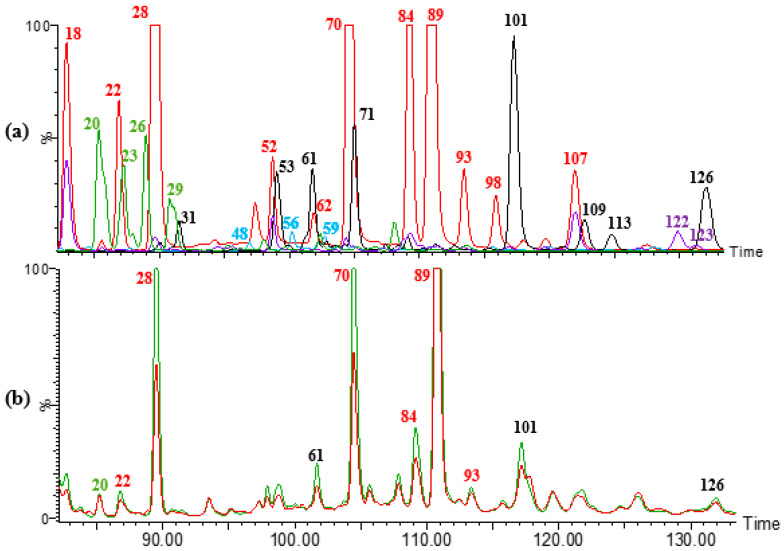
(**a**) Extracted-ion chromatogram (XIC) from 82 to 133 min of dehydrodiferulic acids (DFAs) and dehydrotriferulic acids (TFAs) of maize flour (Verdeal de Aperrela) IF at *m/z* 385 (red), 403 (green), 341 (purple), 577 (black) and 595 (blue). (**b**) Chromatogram of maize flour IF, at 280 nm (red) and 320 nm (green). Peaks are labelled as described in Table 2.

Due to the lack of commercial standards, the identification of DFAs in the samples analysed (maize, wheat, rye, and *broa*) was performed comparing the data obtained with the information described in the literature, when similar analytical conditions were applied, namely: (1) the presence of characteristic precursor ions at *m/z* 385, 403, and 341 [M − H]^−^ (Table 2, Figure 2); (2) MS/MS spectra; (3) characteristic UV absorption spectra with maximum wavelengths at 280 and 320 nm; (4) relative intensities of UV chromatogram peaks at 280 nm; and (5) relative retention times (RTs).

Since DFAs are bound to arabinoxylans [5,6], they were not detected in the SF of any sample (raw flours and *broa*). However, in maize and *broa* IF, 12 peaks at *m/z* 385 were detected and associated with DFAs, according to their characteristic MS/MS spectra. These peaks presented one or more nonspecific product ions at *m/z* 341, 326, 311, 297, 282, and 267, which can be related to the loss of CO_2_ (×2), CH_3_^•^ and CH_2_O, as reported by Callipo et al. [26]. Although only six DFAs with a molecular mass of 386 Da have been commonly described in maize grains, it is known that they can be present as *trans-* or *cis-* isomeric forms, as well as in *anti*- or *syn*- cyclic forms, therefore increasing the number of compounds that can be identified [26].

Dobberstein and Bunzel [24] presented the UV chromatogram at 280 nm of an insoluble fibre extract from whole maize grains and the UV spectra of each isolated DFA [24]. The chromatographic profile obtained in the present work for maize and *broas* IF (Figure 2b) was similar to the one presented by these authors [24]. The main peaks observed at 280 nm and at *m/z* 385 were peaks 28, 70, 84 and 89. According to their MS/MS [26,28,33,38] and UV [24] spectra, relative peaks intensity [24] and RTs [24], they were putatively identified as 8-5′-DFA, 5-5′-DFA, 8-5′-DFA*_f_*, and 8-O-4′-DFA, respectively. These four main DFAs were also detected in rye and wheat IF. Compounds containing an 8-5′(noncyclic)-coupled dimer unit, probably do not exist in plants [4] but are formed from their phenylcoumaran precursors, containing an 8-5′-(cyclic)-coupled dimeric unit, during saponification [4].

The identification of other minor DFAs became a challenge since they have not been so commonly characterised. Peaks 18, 22, and 52 (*m/z* 385) were putatively identified as 8-8′-DFA*_c_,* 8-8′-DFA, and 4-O-5′-DFA, respectively, according to their UV [24] and MS/MS [24,26,33,39] spectra, and elution order [24,33,39].

Additionally, the MS/MS spectra of compounds 93 and 89 (*m/z* 385, 8-O-4′-DFA) were identical. Therefore, compound 89 could correspond to the more common *trans/trans* isomeric form of 8-O-4′-DFA and compound 93 to its *trans/cis* isomeric form [26,33]. Similar to *trans*-8-O-4′-DFA (89), peak 93 showed a characteristic product ion at *m/z* 193, which corresponds to the cleavage of the ether link between the two monomeric units and elimination of a neutral FA, suggesting the presence of a C-O bond, less stable than C-C linkages [33,40]. Furthermore, the characteristic fragments of FA at *m/z* 134, 149 and 178 were also present.

It was possible to detect four additional peaks (49, 62, 98, and 107: DFA 1, 2, 3, and 4) at *m/z* 385 with characteristic DFAs product ions. These compounds may correspond to different isomeric forms of the DFAs already described above, particularly to the *cis* and *syn* configurations.

Another common DFA in maize grains is 8-8′-DFA*_f_* (*m/z* 403). Taking into account its elution order [24,33], and UV [24] and MS/MS [26,33] spectra, this compound was putatively identified as peak 26. Additionally, it was possible to detect three peaks at *m/z* 403 (peaks 20, 23, and 29), with MS/MS spectra related to hydrated forms of DFAs. Another structure related to DFAs is the 8-5′ decarboxylated form (8-5′-DFA*_dc_*) [24,39], with a molecular mass of 342 Da. It was possible to detect peaks 122 and 123 which, according to their elution order [24,26,39], may correspond to *trans*-8-5′-DFA_dc_ and *cis*-8-5′-DFA_dc_, respectively. However, due to their low intensities, it was not possible to compare their UV and MS/MS spectra with data from the literature. It has been described that both compounds are not present in the plant, but instead, they may be formed during the saponification process [4], as previously mentioned for compounds containing an 8-5′(noncyclic)-coupled dimer unit.

Although it has been described that DFAs exhibit higher antioxidant activity than free FA [17], they are not readily absorbed by the human gastrointestinal (GI) system, since they are covalently bound to indigestible polysaccharides [4,18]. However, DFAs can be released by digestive enzymes or microorganisms in the intestinal lumen and be further absorbed [4,41], or exhibit their beneficial action directly in the GI system [4].

### 3.3. Ferulic Acid Dehydrotrimers and Tetramers

Although MS/MS spectra of dehydrotriferulic acids (TFAs) (Figure S3) have not been so commonly characterised, it was possible to putatively identify eight signals at *m/z* 577 [M − H]^−^ and three at *m/z* 595 [M − H]^−^ (hydrated forms of TFAs) [4] in raw flours and breads IF (Figure 2), using the same criteria described for DFAs. In both maize flour and breads IF, peak 101 was the most intense TFA at *m/z* 577, followed by peaks 126 and 71. According to their MS/MS spectra, relative intensities [24,39], and elution order [24,39], they were tentatively identified as 8-O-4′/5-5″-, 8-O-4′/4-O-8″-, and 8-8′*_c_*/4-O-8″-TFA, respectively.

The MS/MS spectrum of peak 101 (8-O-4′/5-5″TFA) showed product ions at *m/z* 533 [M − H − CO_2_]^−^, 489 [M − H − 2CO_2_]^−^, 355, 311, and 193. The detected ion at *m/z* 193 confirmed the presence of a C-O bond, as previously mentioned for DFAs. The signals at *m/z* 355 and 311 could be originated by the fragmentation of the C-O bond and the loss of CH_2_O (−30 Da, *m/z* 355) and CH_2_O and CO_2_ (−74 Da, *m/z* 311) of the 5-5″-diferuloyl moiety. The MS/MS spectrum of peak 126 (8-O-4′/4-O-8″-TFA) showed the presence of the characteristic product ion at *m/z* 193. Fragments with higher *m/z* values, characteristic of C-C linkages, were not detected. Peak 71 (8-8′*_c_*/4-O-8″-TFA) showed a product ion at *m/z* 341 (loss of one feruloyl moiety and CO_2_) and 297 (loss of another CO_2_). The product ion at *m/z* 297 has been described as characteristic of 8-8′-DFA*_c_* [26].

Peak 53 was tentatively identified as 8-O-4′/5-8″-TFA, according to its elution order [39] and MS/MS spectrum, which showed a very intense peak at *m/z* 193, and smaller peaks at *m/z* 355 and 311, similarly to those described for peak 101 (8-O-4′/5-5″-TFA).

Compounds 31, 61, 109, and 113 (TFA 1, 2, 3, and 4) also presented a precursor ion at *m/z* 577. Other minor TFAs that have been described in maize are 8-O-4′/5-8″_C_, 8-8′/4-O-8″-and, possibly, 5-5′/8-8″-TFA [4,42]. Additionally, TFAs may also exhibit *cis* or *trans* configurations, therefore increasing their structural diversity.

A precursor ion at *m/z* 595 was detected in peaks 48, 56, and 64 (TFA, hydrated 1, 2, and 3), which may correspond to isomers of 8-8′*_f_*/5-5″-TFA or 8-O-4′/5-5″(H_2_O)-TFA.

Dehydrotetraferulic acids (TeFA) have also been reported in maize bran [4]. The extracted ion chromatogram (XIC) at *m/z* 769 showed a chromatogram with several low-intensity peaks. According to their elution order, two of them, peak 94 and peak 130, may correspond to 4-O-8′/5′-5″/8″-5″′-TeFA and 4-O-8′/5′-5″/8″-O-4″′-TeFA, respectively (Figure S4).

Similar to FA dehydrodimers, FA trimers and tetramers are not readily absorbed by the human GI system and need to be released by digestive enzymes or microorganisms in the intestinal lumen before absorption [4]. However, since they were also detected in *broa*, they should be considered when studying the phenolic composition of maize-based food products and in bioavailability studies.

### 3.4. Soluble Hydroxycinnamic Acid Amides

**Figure 3:**
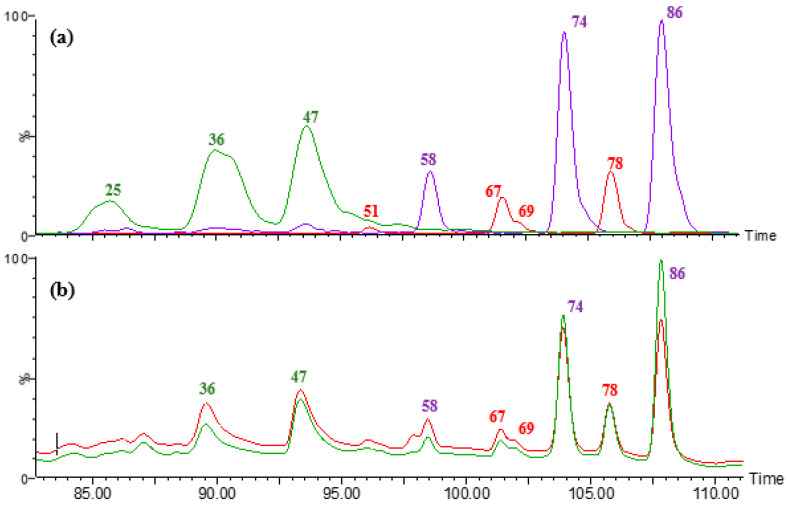
(**a**) Extracted-ion chromatogram (XIC) of hydroxycinnamic acid amides (HCAAs) of *broa* Verdeal de Aperrela SF, at *m/z* 411 (red), 438 (green), and 441 (purple). (**b**) Chromatogram of *broa* SF, at 280 nm (red) and 320 nm (green). Peaks are labelled as described in Table 2.

The major peaks observed in the SF of maize flours and *broas* (Figure 3) were peaks 51, 67, 69, and 78 at *m/z* 409 or 411, peaks 25, 36, and 47 at *m/z* 436 or 438, and peaks 58, 74, and 86 at *m/z* 439 or 441 ([M −− H]^−^ or [M + H]^+^, respectively), corresponding to the monoisotopic masses (MM) of 410, 437 and 440. These compounds were tentatively identified as HCAAs, namely *N,N*′-coumaroyl feruloyl putrescine (CFP), *N,N*′-dicoumaroyl spermidine (DCS), and *N,N*′-diferuloyl putrescine (DFP) (Figure S5, Table 2), which have not been described in maize-based foods before. HCAAs are formed by hydroxycinnamic moieties in which double bonds can assume either a *cis* or *trans* configuration [11], giving rise to the formation of several isomeric amides with the same precursor and product ions. The different isomers observed in the present work correspond to the different possibilities of double bonds configuration on FA and pCA moieties. In plants, the *cis* isomers are less common [4] and, in reversed-phase chromatography, they elute earlier than *trans* isomers [31], presenting lower peak areas as well (Figure 3). These compounds were not detected in neither the analysed wheat nor rye flours. Indeed, it is known that maize contains large amounts of conjugated putrescine and spermidine, when comparing to other cereals, such as rice and wheat [31].

The monoconjugate *N*-coumaroyl spermidine (21) was identified in *broas*, probably formed from the hydrolysis of DCS during processing. The monoconjugates feruloyl and *p*-coumaroyl putrescine were not detected either in maize flours or *broas*, although they have been described in maize grains [31,43]. A possible explanation could be that the extraction procedures used by other authors (80% of methanol with 1% of HCl and methanol/isopropanol/water, 8/1/1), could have led to the hydrolysis of more complex HCAAs, liberating the described monoconjugates.

Other minor HCAAs were also identified in maize flours and *broas* extracts, namely compounds 32, 40, 46, 54 (*N,N′*-coumaroyl feruloyl spermidine isomers, CFS), compounds 44, 60, 73 (*N,N′*-dicoumaroyl putrescine isomers, DCP), and compounds 39, 45, 57 (*N,N′*-diferuloyl spermidine isomers, DFS). Compounds 40 and 46 (CFS) were also detected in the commercial wheat and rye flours SF and compounds 39 and 57 (DFS) were also detected in wheat flour SF. These compounds have been recently described in maize grains [31].

Peak 82 showed a precursor ion at *m/z* 877 [M − H]^−^, with a UV spectrum similar to other HCAAs (λmax: 316, 293) and was detected in maize flours and *broas* extracts. The MS/MS experiments showed a product ion at *m/z* 439, which may correspond to a DFP molecule. This compound was putatively identified as *bis*-*N,N′*-diferuloyl putrescine, a molecule with two DFP moieties. To the best of our knowledge, this compound has never been previously described.

Recently, some studies have pointed out that HCAAs, specially feruloyl putrescines, exhibit antioxidant [7,8], anti-inflammatory [44] and chemopreventive [13] properties, capable of inducing apoptosis in human leukemia U937 cells [14]. However, these compounds have not been studied in maize-based foods and thus there is no information about their bioavailability. HCAAs were detected in the SF of *broas*, which suggests that they can be readily absorbed or easily exposed to the action of digestive enzymes [4].

### 3.5. Insoluble Hydroxycinnamic Acid Amides

Some of the HCAAs described above were also identified in the IF of maize flours and *broas* (Table 2). Therefore, they were probably linked to the maize grain matrix, such as cell walls, as previously suggested [11]. Analyses by XIC in positive ion mode were performed in order to search for the presence of dehydrodiferulic and dehydrotriferulic acid amides. Results suggest that these compounds were present in maize flours and *broas* IF, but not in wheat or rye flours. To the best of our knowledge, these compounds have not been previously described, and these results provide evidence that HCAAs are also constituents of maize cell walls.

**Figure 4:**
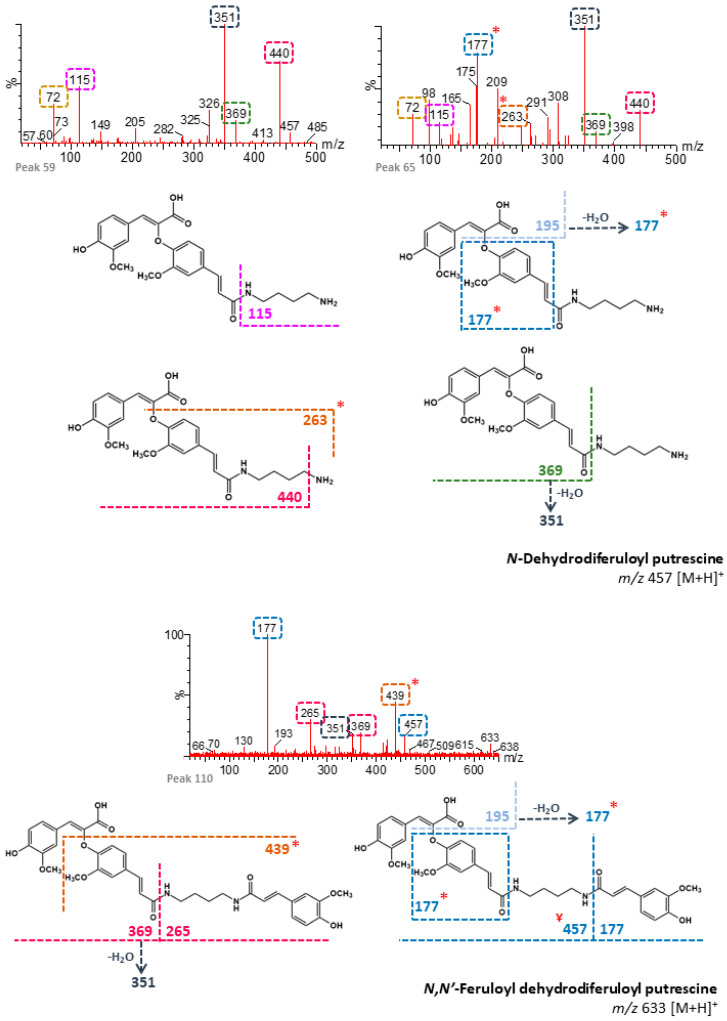

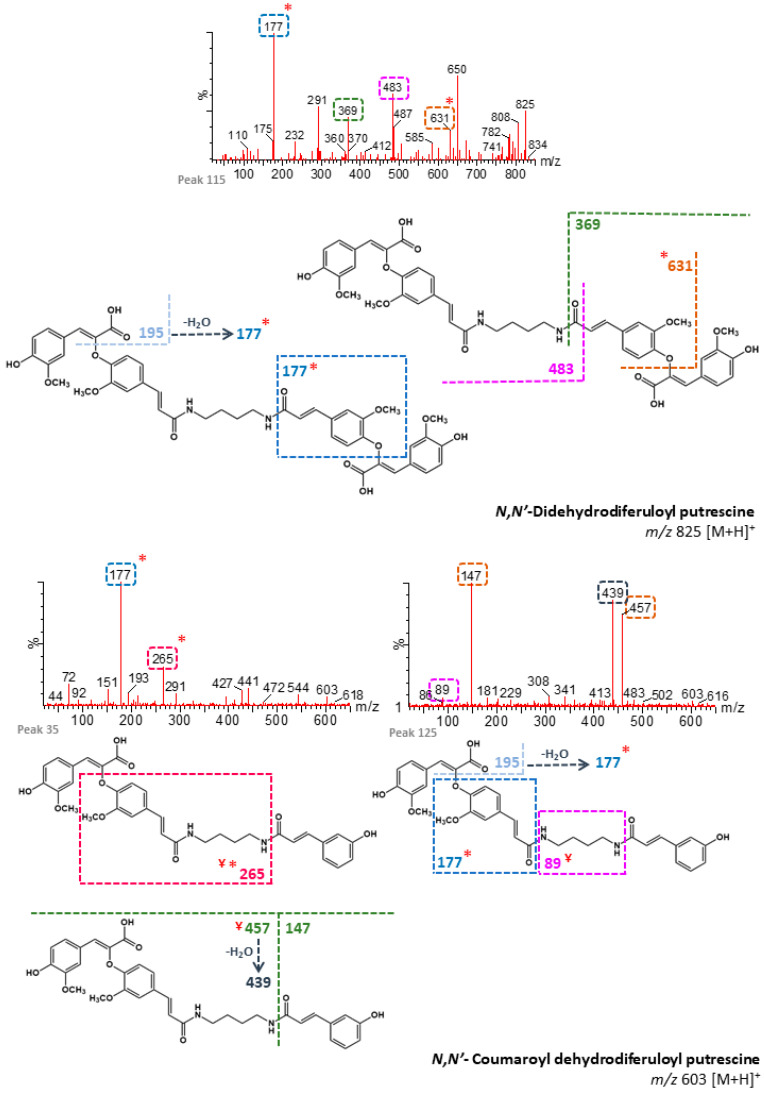
Fragmentation patterns proposed for dehydrodiferuloyl (8-O-4′-DFA) putrescines. *: Characteristic product ions of C-O linkages between feruloyl moieties. **^Ұ^**: Product ions formed after amine protonation.

**Figure 5:**
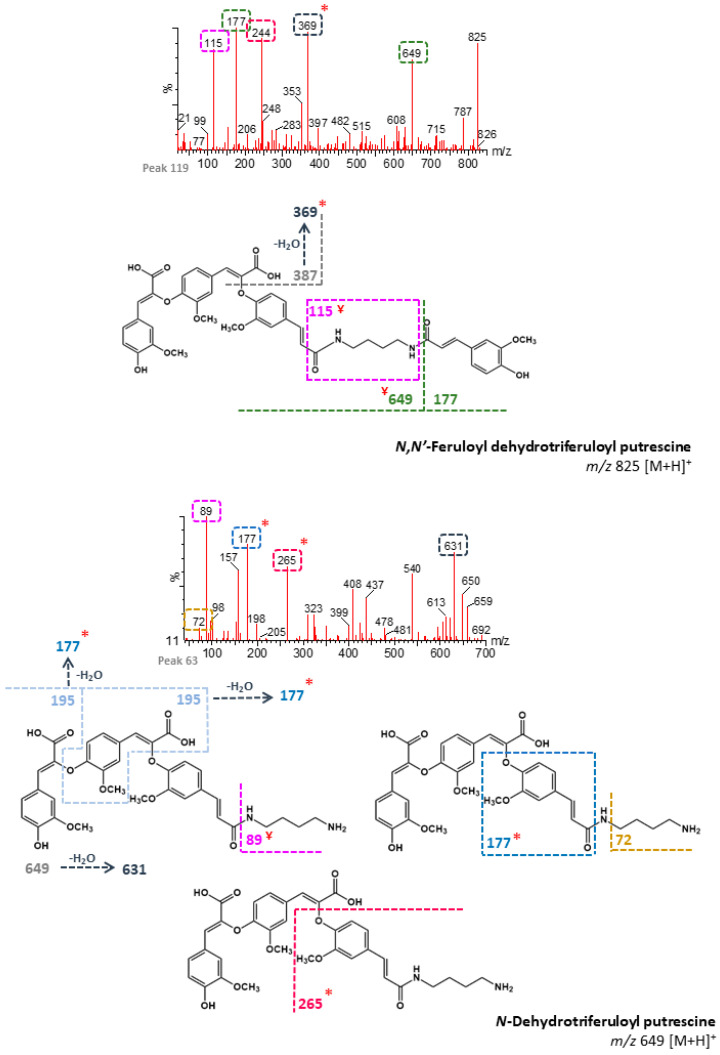

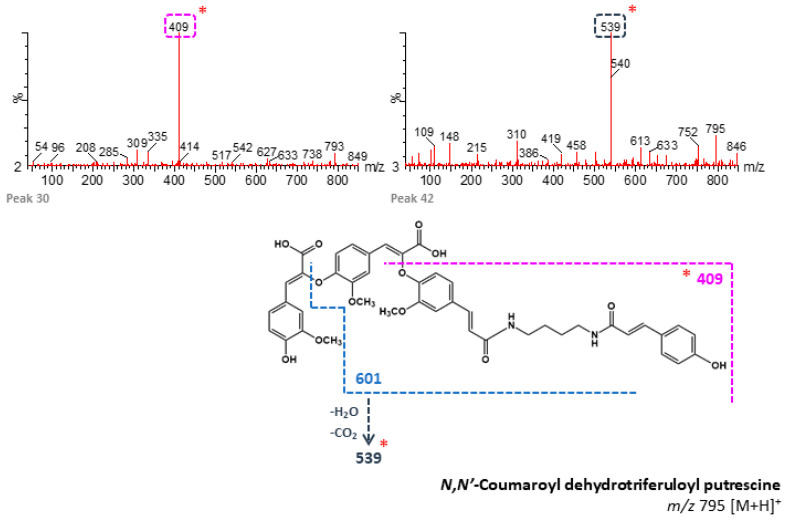
Fragmentation patterns proposed for dehydrotriferuloyl (8-O-4′/4-O-8′’-TFA) putrescines. *: Characteristic product ions of C-O linkages between feruloyl moieties. **^Ұ^**: Product ions formed after amine protonation.

Since there are several isomeric forms of DFAs, numerous dehydrodiferuloyl and dehydrotriferuloyl putrescine isomers can also be formed. Seven peaks were tentatively identified as *N*-dehydrodiferuloyl (*m/z* 457, [M + H]^+^), twenty-five as *N,N′*-feruloyl dehydrodiferuloyl (*m/z* 633), four as *N,N′*-didehydrodiferuloyl (*m/z* 825), and eleven as *N,N′*-coumaroyl dehydrodiferuloyl (*m/z* 603) putrescines (Figure S5). Additionally, nine peaks were tentatively identified as *N*-dehydrotriferuloyl (*m/z* 649), two as *N,N′*-feruloyl dehydrotriferuloyl (*m/z* 825), and seven as *N,N′*-coumaroyl dehydrotriferuloyl (*m/z* 795) putrescines (Figure S5). Figure 4 and Figure 5 show the fragmentation patterns proposed for several dehydrodiferulic acid and dehydrotriferuloyl putrescines, respectively. These compounds were identified mainly based on the characteristic cleavages between amide bonds that have been described for HCAAs [31], which originate, among others, from the product ions coumaroyl (*m/z* 147), feruloyl (*m/z* 177), and dehydrodiferuloyl (*m/z* 369). Feruloyl dehydrotriferuloyl putrescines were distinguished from didehydrodiferuloyl putrescines (*m/z* 825) by the presence of the product ion at *m/z* 649 (dehydrotriferuloyl putrescine) (Figure 5). According to the chemical structures proposed for each dehydrodiferulic and dehydrotriferulic acid putrescines, specific product ions suggest the presence of C-O linkages between feruloyl moieties, probably 8-O-4′ linkages. In contrast, the absence of these ions suggests the presence of C-C linkages. However, for simplification purposes, only 8-O-4′ linkages are represented in Figure 4 and Figure 5. It was not possible to detect either HCAAs derived from hydrated forms of DFAs or TFAs, nor spermidine-linked DFAs or TFAs.

Since it has been described that HCAAs and FA dehydrodimers exhibit higher antioxidant and anti-inflammatory activities than free FA [8,17,44], insoluble HCAAs constituted by FA dehydrodimers and trimers may also exhibit interesting beneficial health effects. Future studies on the bioactivity of these compounds should be performed. As FA dehydrodimers, trimers and tetramers, insoluble HCAAs were only detected after hydrolysis, which suggests they were bound to indigestible polysaccharides, and therefore are not easily absorbed by the human GI system, but can eventually exhibit their action in this system.

## 4. Conclusions

This study sheds light on the identification of different isomers of FA dimers, FA trimers and HCAAs in maize flour and *broas*, by HPLC-DAD-MS/MS analysis. Complex HCAAs were identified for the first time, consisting of putrescine-linked DFAs and TFAs, suggesting that HCAAs are associated with maize cell walls. The presence of these compounds in *broas* shows that they are resistant to processing. Thus, they can also contribute to the total phenolic content and antioxidant properties of this maize-based bread and associated health benefits. Therefore, in addition to FA and pCA, hydroxycinnamic acid derivatives should be considered when studying the phenolic composition of maize and maize-based food products. Future work is needed in order to characterise more isomeric forms of DFAs, TFAs, and their respective putrescine derivatives using mass spectrometry tools. Additionally, the intestinal release and uptake of the studied compounds, especially soluble HCAAs, should be evaluated. The differences among the phenolics content of raw flours and *broas* are currently being studied, to understand the possible contribution of *broa* as a health-promoting bread.

## Figures and Tables

**Table 1:** Description of the maize varieties or flour used in the production of *broas.*

Variety/Flour	Description
Broa-213	Yellow grain. Early intermediate type. Collect from the farmer in the 2005 expedition to the Central Northern region of Portugal [20].
Pigarro	White grain. Flint type FAO 300 with strong fasciation expression, used in the best soils for human consumption. Participatory improved population [20].
Castro Verde	Yellow grain. Late flint type FAO 600, with big grain row number and large ear size. Participatory improved population [20].
Verdeal de Aperrela	White grain. Late flint type FAO 600 used for breadmaking. Participatory improved population [20].
Fandango	Yellow grain. Synthetic open-pollinated variety, dent type FAO 600, big grain row number and large ear size. Participatory improved population [20].
Commercial	White colour. Nacional Type 175, wholegrain flour.

**Table 2:** Compounds identified by HPLC-DAD-MS/MS (high-performance liquid chromatography coupled with diode array detector and tandem mass spectrometry) in maize (Verdeal de Aperrela), wheat, and rye flours and *broa* prepared from Verdeal de Aperrela maize flour, in the soluble (SF) and insoluble (IF) fractions.

#	λmax	RT	Putative Identification	*m/z*	MS/MS Ions	SF	IF
Small Phenolic Compounds							
3	260, 294	30.96	Protocatechuic acid ^(−) Ұ^	153	109, 108	-	B, M, R, W
4	286	39.65	Ferulic acid hexoside 1 ^(+)^	357	**195**	B	-
5	n/d	46.16	Ferulic acid hexoside 2 ^(+)^	357	**195**, 149, 185	B	-
6	325, 298	46.93	Vanillic acid ^(−) Ұ^	169	93, 123, 65	B, M, R, W	B, M, R, W
7	294	47.83	Ferulic acid hexoside 3 ^(+)^	357	**195**, 149, 185	B, W	-
8	324, 295	51.99	Caffeic acid ^(−) Ұ^	179	135	B	B, M, R, W
9	275	53.60	Syringic acid ^(+) Ұ^	199	140, 155, 123	B, M, R, W	B, M, R, W
10	286	55.08	*p*-Hydroxybenzaldehyde ^(−)^	121	39, 92	B, M, R, W	B, M, R, W
11	279, 309	65.88	Vanillin ^(+) Ұ^	153	93, 125, 65	B, M, R, W	B, M, R, W
12	296	69.60	Coumaroyl glycerol ^(−)^	237	**145**, **119**, **163**	B, M, R	-
13	310	70.91	*p*-Coumaric acid (*trans*) ^(−) Ұ^	163	119, 93	B, M, R, W	B, M, R, W
14	297	71.31	Syringaldehyde ^(+) Ұ^	183	123, 95, 155, 140	B, M, R	B, M, R, W
15	n/d	73.00	*p*-Coumaric acid (*cis*) ^(−)^	163	119, 93	B, M, R, W	B, M, R, W
16	322, 295	78.97	Ferulic acid (*trans*) ^(−) Ұ^	193	134, 149, 178	B, M, R, W	B, M, R, W
17	312	81.11	Ferulic acid (*cis*) ^(−)^	193	134, 149, 178	B, M, R, W	B, M, R, W
Ferulic Acid Dehydrodimers							
18	333	82.86	8-8′-DFA*_c_*^(−)^	385	267, **158**, **173**	-	B, M, R, W
20	318, 284	85.29	DFA, hydrated 1 ^(−)^	403	178, 148, 193, 134	-	B, M, R, W
22	325	86.84	8-8′-DFA ^(−)^	385	282, **173**, **123**	-	B, M, R
23	n/d	87.23	DFA, hydrated 2 ^(−)^	403	239, 279, 265, 134, 148	-	B, M
26	284, 318	88.91	8-8′-DFA*_f_*^(−)^	403	**151**, 148, **233**, **163**	-	B, M, R
28	322	89.64	8-5′-DFA ^(−)^	385	**282**, **267**, **326**, **297**, 323, **341**	-	B, M, R, W
29	n/d	90.88	DFA, hydrated 3 ^(−)^	403	193, 308, 149, 164	-	B, M, R
49	284, 318	97.33	DFA 1 ^(−)^	385	173, 123, 282	-	B, M
52	322	98.64	4-O-5′-DFA ^(−)^	385	139, **193**, 267, 329	-	B, M
62	318	101.78	DFA 2 ^(−)^	385	267, 382	-	B, M, R, W
70	322	104.50	5-5-DFA ^(−)^	385	**282**, **326**, **341**, **267**	-	B, M, R, W
84	319	109.19	8-5′-DFA*_f_* ^(−)^	385	**282**, **326**, **341**, **267**	-	B, M, R, W
89	322, 294	110.85	8-O-4-DFA (*trans/trans*) ^(−)^	385	**134**, **178**, **149**, **193**	-	B, M, R, W
93	318, 289	113.35	8-O-4-DFA (*trans/cis*) ^(−)^	385	**134**, **178**, **149**, **193**	-	B, M
98	318	115.83	DFA 3 ^(−)^	385	-	-	B, M, R
107	318	121.94	DFA 4 ^(−)^	385	-	-	B, M
122	284	129.81	8-5′-DFA*_dc_* (*trans*) ^(−)^	341	-	-	B, M
123	n/d	131.10	8-5′-DFA*_dc_* (*cis*) ^(−)^	341	-	-	B, M
Ferulic Acid Dehydrotrimers and Tetramers							
31	n/d	91.54	TFA 1 ^(−)^	577	435, 508, 178	-	B, M, R
48	n/d	96.94	TFA, hydrated 1 ^(−)^	595	-	-	B, M
53	322	98.91	8-O-4′/5-8″-TFA ^(−)^	577	193, 355	-	B, M, R
56	318	100.12	TFA, hydrated 2 ^(−)^	595	317, 545, 367	-	B, M
61	318	101.70	TFA 2 ^(−)^	577	146	-	B, M
64	316	102.67	TFA, hydrated 3 ^(−)^	595	-	-	B, M
71	n/d	104.91	8-8′*_c_*-/4-O-8″-TFA ^(−)^	577	341, **533**, **489**	-	B, M
94	318	113.54	4-O-8′/5′-5″/8″-5″′-TeFA ^(−)^	769	274	-	B, M
101	319	117.17	8-O-4′-5-5″-TFA ^(−)^	577	355, **533**, **193**, **489**	-	B, M, R, W
109	318	122.74	TFA 3 ^(−)^	577	355	-	B, M
113	316	124.73	TFA 4 ^(−)^	577	355	-	B, M
126	319, 291	131.98	8-O-4′/4-O-8″-TFA ^(−)^	577	**193**	-	B, M, R
130	318	135.24	4-O-8′/5′-5″/8″-O-4-TeFA ^(−)^	769	**193**	-	B, M
Soluble Hydroxycinnamic Acid Amides							
21	290	85.88	*N*-Coumaroyl spermidine ^(+)^	292	**147**, 204	B	-
25	n/d	88.74	*N,N′*-Dicoumaroyl spermidine (*cis/cis*) ^(+)^	438	**147**, 204, **292**, 275, 72	B, M	B, M
32	n/d	91.75	*N,N′*-Coumaroyl feruloyl spermidine (*cis/cis*) ^(+)^	468	**177**, 234, 204, **292**, **147**, **322**, 145	B, M	-
36	290	93.05	*N,N′*-Dicoumaroyl spermidine (*cis/trans*) ^(+)^	438	**147**, 204, **292**, 275, 72	B, M	B, M
39	n/d	94.40	*N,N′*-Diferuloyl spermidine (*cis/cis*) ^(+)^	498	**177**, 234, 322, 145	B, M, W	-
40	n/d	94.60	*N,N′*-Coumaroyl feruloyl spermidine (*cis/trans*) 1 ^(+)^	468	**177**, 204, **292**, **147**, **322**, 234, 275, 145	B, M, W, R	-
44	n/d	96.13	*N,N′*-Dicoumaroyl putrescine (*cis/cis*) ^(+)^	381	189, 145, **147**, 101, 277, 177, 321	B, M	-
45	n/d	96.42	*N,N′*-Diferuloyl spermidine (*cis/trans*) ^(+)^	498	**177**, 234, 322, 305, 145	B, M	-
46	n/d	96.59	*N,N′*-Coumaroyl feruloyl spermidine (*cis/trans*) 2 ^(+)^	468	**177**, 204, **292**, **147**, **322**, 234, 275, 145	B, M, W, R	-
47	294	96.62	*N,N′*-Dicoumaroyl spermidine (*trans/trans*) ^(+)^	438	**147**, 204, **292**, 275, 72, 221	B, M	B, M
51	n/d	98.52	*N,N′*-Coumaroyl feruloyl putrescine (*cis/cis*) ^(+)^	411	**177**, **147**, **235**	B, M	B, M
54	n/d	99.44	*N,N′*-Coumaroyl feruloyl spermidine (*trans/trans*) ^(+)^	468	**177**, 234, 204, **292**, **322**, **147**, 145, 305, 275	B, M	-
57	n/d	100.61	*N,N′*-Diferuloyl spermidine (*trans/trans*)	498	**177**, **322**, 234, 145,	B, M, W	-
58	290	100.83	*N,N′*-Diferuloyl putrescine (*cis/cis*) ^(+)^	441	**177**, 265, 145, 89, 117, 248	B, M	B, M
60	n/d	101.43	*N,N′*-Dicoumaroyl putrescine (*cis/trans*) ^(+)^	381	**147**, **235**, 218	B, M	-
67	291	103.47	*N,N′*-Coumaroyl feruloyl putrescine (*cis/trans*) 1 ^(+)^	411	**177**, **147**, 145, 265, **235**, 218	B, M	B, M
69	292	104.09	*N,N′*-Coumaroyl feruloyl putrescine (*cis/trans*) 2 ^(+)^	411	**177**, **147**, 145, 265, **235**, 218	B, M	B, M
73	n/d	105.30	*N,N′*-Dicoumaroyl putrescine (*trans/trans*) ^(+)^	381	**147**, **235**, 218, 89, 72	B, M	-
74	293	105.88	*N,N′*-Diferuloyl putrescine (*cis/trans*) ^(+)^	441	**177**, 145, 265, 248	B, M	B, M
78	292, 308	107.56	*N,N′*-Coumaroyl feruloyl putrescine (*trans/trans*) ^(+)^	411	**177**, **147**, 145, 235, 265, 89, 218	B, M	B, M
82	290	108.65	*bis-N,N′*-Diferuloyl putrescine ^(−)^	877	439	B, M	-
86	317, 293	109.65	*N,N′*-Diferuloyl putrescine (*trans/trans*) ^(+)^	441	177, 145, 265, 248, 89, 117, 72	B, M	B, M
Insoluble Hydroxycinnamic Acid Amides							
19	n/d	84.03	*N,N′*-Coumaroyl dehydrotriferuloyl putrescine 1 ^(+)^	795	409, 519, 719	-	M
24	n/d	87.48	*N,N′*-Coumaroyl dehydrodiferuloyl putrescine 1 ^(+)^	603	177, 427, 265, 195	-	B, M
27	n/d	89.40	*N,N′*-Coumaroyl dehydrodiferuloyl putrescine 2 ^(+)^	603	177, 265	-	B, M
30	n/d	91.20	*N,N′*-Coumaroyl dehydrotriferuloyl putrescine 2 ^(+)^	795	409	-	B, M
33	n/d	92.22	*N*-Dehydrodiferuloyl putrescine 1 ^(+)^	457	89, 72, 115	-	B, M
34	n/d	92.24	*N,N′*-Coumaroyl dehydrotriferuloyl putrescine 3 ^(+)^	795	409, 533	-	B, M
35	n/d	92.44	*N,N′*-Coumaroyl dehydrodiferuloyl putrescine 3 ^(+)^	603	177, 265, 72	-	B, M
37	n/d	93.35	*N*-Dehydrodiferuloyl putrescine 2 ^(+)^	457	115, 265, 72	-	B, M
38	n/d	93.59	*N,N′*-Coumaroyl dehydrodiferuloyl putrescine 4 ^(+)^	603	415, 148	-	B, M
41	n/d	95.02	*N*-Dehydrodiferuloyl putrescine 3 ^(+)^	457	115, 298, 72	-	B, M
42	n/d	95.61	*N,N′*-Coumaroyl dehydrotriferuloyl putrescine 4 ^(+)^	795	539, 148	-	B, M
43	n/d	95.68	*N*-Dehydrodiferuloyl putrescine 4 ^(+)^	457	351, 319, 277, 115	-	B, M
50	n/d	97.44	*N,N′*-Coumaroyl dehydrotriferuloyl putrescine 5 ^(+)^	795	539, 135, 195	-	B, M
55	n/d	99.70	*N*-Dehydrodiferuloyl putrescine 5 ^(+)^	457	115, 98, 177, 244, 365	-	B, M
59	n/d	101.10	*N*-Dehydrodiferuloyl putrescine 6 ^(+)^	457	351, 440, 115, 72, 369	-	B, M
63	n/d	101.80	*N*-Dehydrotriferuloyl putrescine 1 ^(+)^	649	89, 177, 631, 265, 72	-	B, M
65	n/d	103.11	*N*-Dehydrodiferuloyl putrescine 7 ^(+)^	457	351, 177, 175, 440, 72, 115, 263	-	B, M
66	n/d	103.33	*N,N′*-Coumaroyl dehydrotriferuloyl putrescine 6 ^(+)^	795	539, 394, 435		B, M
68	n/d	103.87	*N,N′*-Feruloyl dehydrodiferuloyl putrescine 1 ^(+)^	633	177, 432, 465, 387	-	B, M
72	n/d	105.27	*N,N′*-Feruloyl dehydrodiferuloyl putrescine 2 ^(+)^	633	177, 457, 369, 341, 72	-	B, M
75	n/d	106.33	*N,N′*-Coumaroyl dehydrodiferuloyl putrescine 5 ^(+)^	603	457, 369, 72, 83, 369, 411	-	B, M
76	n/d	106.82	*N,N′*-Feruloyl dehydrodiferuloyl putrescine 3 ^(+)^	633	177, 265, 439, 457	-	B, M
77	n/d	107.45	*N,N′*-Feruloyl dehydrodiferuloyl putrescine 4 ^(+)^	633	177, 341, 439, 589	-	B, M
79	n/d	107.97	*N*-Dehydrotriferuloyl putrescine 2 ^(+)^	649	265, 89, 177, 440	-	B, M
80	n/d	108.18	*N,N′*-Feruloyl dehydrodiferuloyl putrescine 5 ^(+)^	633	177, 369, 457, 439, 574, 291, 145, 89	-	B, M
81	n/d	108.58	*N,N′*-Coumaroyl dehydrodiferuloyl putrescine 6 ^(+)^	603	457, 385	-	B, M
83	n/d	109.16	*N*-Dehydrotriferuloyl putrescine 3 ^(+)^	649	89, 72, 148, 265	-	B, M
85	n/d	109.34	*N,N′*-Feruloyl dehydrodiferuloyl putrescine 6 ^(+)^	633	177, 351, 245, 439	-	B, M
87	n/d	110.28	*N,N′*-Coumaroyl dehydrodiferuloyl putrescine 7 ^(+)^	603	365, 439, 351	-	B, M
88	n/d	110.34	*N,N′*-Feruloyl dehydrodiferuloyl putrescine 7 ^(+)^	633	457, 177, 439, 369, 265	-	B, M
90	n/d	110.90	*N,N′*-Coumaroyl dehydrotriferuloyl putrescine 7 ^(+)^	795	409	-	B, M
91	n/d	111.80	*N,N′*-Feruloyl dehydrodiferuloyl putrescine 8 ^(+)^	633	177, 439, 457, 589	-	B, M
92	n/d	112.32	*N,N′*-Feruloyl dehydrodiferuloyl putrescine 9 ^(+)^	633	265, 177, 351, 369, 439	-	B, M
95	n/d	113.60	*N*-Dehydrotriferuloyl putrescine 4 ^(+)^	649	72, 89, 177, 631	-	B, M
96	n/d	114.02	*N,N′*-Feruloyl dehydrodiferuloyl putrescine 10 ^(+)^	633	439, 457, 369, 277	-	B, M
97	n/d	115.02	*N,N′*-Feruloyl dehydrodiferuloyl putrescine 11 ^(+)^	633	519, 439, 351, 145, 175, 177	-	B, M
99	n/d	115.88	*N*-Dehydrotriferuloyl putrescine 5 ^(+)^	649	177, 115	-	B, M
100	n/d	116.69	*N,N′*-Feruloyl dehydrodiferuloyl putrescine 12 ^(+)^	633	245, 151, 291, 351, 177, 439	-	B, M
102	n/d	117.21	*N*-Dehydrotriferuloyl putrescine 6 ^(+)^	649	245, 177, 323	-	B, M
103	n/d	118.49	*N,N′*-Feruloyl dehydrodiferuloyl putrescine 13 ^(+)^	633	439, 457, 177	-	B, M
104	n/d	118.97	*N,N′*-Didehydrodiferuloyl putrescine 1 ^(+)^	825	367, 631	-	B, M
105	n/d	119.43	*N,N′*-Feruloyl dehydrodiferuloyl putrescine 14 ^(+)^	633	177, 439, 351, 457, 115, 89	-	B, M
106	n/d	120.04	*N,N′-*Feruloyl dehydrotriferuloyl putrescine 1 ^(+)^	825	631, 177, 265, 649	-	B, M
108	n/d	122.23	*N*-Dehydrotriferuloyl putrescine 7 ^(+)^	649	382, 265, 72, 89, 439	-	B, M
110	n/d	122.80	*N,N′*-Feruloyl dehydrodiferuloyl putrescine 15 ^(+)^	633	177, 439, 265, 369, 351, 457	-	B, M
111	n/d	123.55	*N*-Dehydrotriferuloyl putrescine 8 ^(+)^	649	473, 145, 177	-	B, M
112	n/d	124.17	*N,N′*-Feruloyl dehydrodiferuloyl putrescine 16 ^(+)^	633	351, 177, 265, 72	-	B, M
114	n/d	124.93	*N,N′*-Coumaroyl dehydrodiferuloyl putrescine 8 ^(+)^	603	457, 235, 147	-	B, M
115	n/d	125.38	*N,N′*-Didehydrodiferuloyl putrescine 2 ^(+)^	825	177, 650, 483, 369, 631	-	B, M
116	n/d	126.08	*N,N′*-Coumaroyl dehydrodiferuloyl putrescine 9 ^(+)^	603	439, 369, 147, 457	-	B, M
117	n/d	126.54	*N,N′*-Feruloyl dehydrodiferuloyl putrescine 17 ^(+)^	633	177, 457, 439	-	B, M
118	n/d	127.85	*N,N′*-Feruloyl dehydrodiferuloyl putrescine 18 ^(+)^	633	177, 369, 265, 115, 439	-	B, M
119	n/d	127.97	*N,N′-*Feruloyl dehydrotriferuloyl putrescine 2 ^(+)^	825	177, 369, 244, 115, 649	-	B, M
120	n/d	129.06	*N,N′*-Coumaroyl dehydrodiferuloyl putrescine 10 ^(+)^	603	457, 369, 86, 175, 219, 147	-	B, M
121	n/d	129.76	*N,N′*-Feruloyl dehydrodiferuloyl putrescine 19 ^(+)^	633	177, 439, 457, 589, 115, 145	-	B, M
124	n/d	131.12	*N*-Dehydrotriferuloyl putrescine 9 ^(+)^	649	351, 177	-	B, M
125	n/d	131.19	*N,N′*-Coumaroyl dehydrodiferuloyl putrescine 11 ^(+)^	603	147, 439, 457, 89	-	B, M
127	n/d	132.59	*N,N′*-Didehydrodiferuloyl putrescine 3 ^(+)^	825	177, 631	-	B, M
128	n/d	133.20	*N,N′*-Feruloyl dehydrodiferuloyl putrescine 20 ^(+)^	633	177, 369, 457, 265, 291, 439, 145, 72, 89	-	B, M
129	n/d	134.97	*N,N′*-Feruloyl dehydrodiferuloyl putrescine 21 ^(+)^	633	177, 457, 439, 291, 145, 351, 589	-	B, M
131	n/d	139.79	*N,N′*-Feruloyl dehydrodiferuloyl putrescine 22 ^(+)^	633	177, 457, 439	-	B, M
132	n/d	144.90	*N,N′*-Didehydrodiferuloyl putrescine 4 ^(+)^	825	177, 439	-	B, M
133	n/d	145.78	*N,N′*-Feruloyl dehydrodiferuloyl putrescine 23 ^(+)^	633	177, 457, 439, 135	-	B, M
134	n/d	150.15	*N,N′*-Feruloyl dehydrodiferuloyl putrescine 24 ^(+)^	633	177, 457	-	B, M
135	n/d	153.38	*N,N′*-Feruloyl dehydrodiferuloyl putrescine 25 ^(+)^	633	177	-	B, M
Other Compounds							
1	282	11.50	Citric acid ^(−) Ұ^	191	87, 111, 85, 67	B, M, R, W	B, M
2	n/d	28.45	Tyrosyl-tryptophan ^(-)^	366	160	B, M	-

# Peak number; ^(+)^ compounds detected in positive ion mode; ^(−)^ compounds detected in negative ion mode, ^Ұ^ compounds identified by commercial standards; RT: retention time (minutes); SF: soluble fraction; IF: insoluble fraction; B, M, R, W: compounds detected in bread, maize, rye, and wheat; n/d: not determined due to the peak’s low intensity and/or poor peak resolution. Bold: characteristic fragment ions described by other authors [7,9,10,12,13,25,26,27,28,29,30,31,32,33]; main MS/MS ions are ordered according to their decreasing intensities.

## Supplementary Materials

The following are available online at https://www.mdpi.com/2304-8158/9/10/1471/s1, Figure S1: Comparison of chromatographic profiles of (A) maize flours soluble fraction (SF), (B) *broas* SF, (C) maize flour insoluble fraction (IF) and (D) *broas* IF at 280 nm, Figure S2: Chemical structures of the most known dehydrodiferulic acids (DFAs), Figure S3: Chemical structures of the most known dehydrotriferulic acids (TFAs), Figure S4: Chemical structures of the most known dehydrotetraferulic acids (TeFAs), Figure S5: Molecular structures of soluble hydroxycinnamic acid amides (HCAAs), Figure S6: Chemical structures suggested for 8-O-4′-dehydrodiferulic and 8-O-4′/4-O-8″-dehydrotriferulic acid putrescines, Table S1: Details of the MRM conditions applied in the HPLC-DAD-MS/MS analysis.

## Appendix A

The eluents used were A: 0.1% phosphoric acid in Milli-Q^®^ water and B: 0.1% phosphoric acid in acetonitrile and Milli-Q^®^ water (0.1/40/59.9), at a flow rate of 0.7 mL min^−1^. The following gradient of eluents was used: 0–20% B over 15 min, held isocratically at 20% B for 10 min, 20–70% B from 25 to 70 min, 70% B for 5 min, 70–100% B from 75 to 85 min, and finally, 100% B for 15 min, followed by an equilibration step of 10 min. DAD was programmed for scanning between 192 and 798 nm at a speed of 1 Hz with a bandwidth of 5 nm. The detection was monitored using three individual channels, 280, 320 and 360 nm, at a speed of 10 Hz with a bandwidth of 11 nm.

## Appendix B

The mobile phase consisted of water with 0.5% formic acid as eluent A and acetonitrile as eluent B at a flow rate of 0.30 mL min^−1^. The system was run with the following gradient program: 0–15 min from 1 to 10% B; 15–20 min from 10 to 11% B; 20–30 min at 11% B; 30–45 min from 11 to 15% B; 45–55 min at 15% B; 55–95 min from 15 to 30% B; 95–150 min at 30% B; 150–160 min from 30 to 50% B; 160–180 min at 50% B; finally returning to the initial conditions for 20 min. DAD was used to scan wavelength absorption from 210 to 600 nm. Tandem mass spectrometry (MS/MS) detection was performed using an electrospray ionisation (ESI) source operating at 120 °C, applying a capillary voltage of 3.0 kV, cone voltage of 30 V, and collision energies of 10, 20, and 30 eV. The compounds were ionised in both negative and positive ion modes. High purity nitrogen (N_2_) was used both as drying gas and as a nebulising gas. Ultra-high purity Argon (Ar) was used as the collision gas.
