# Supplementary material for: Hydroxycinnamic Acids and Their Derivatives in Broa, a Traditional Ethnic Maize Bread
Source: Foods. 2020 Oct 15;9(10):1471. doi: 10.3390/foods9101471 (PMC7602622; doi:10.3390/foods9101471)
Supplement: Supplementary file 1 [file foods-09-01471-s001.pdf]

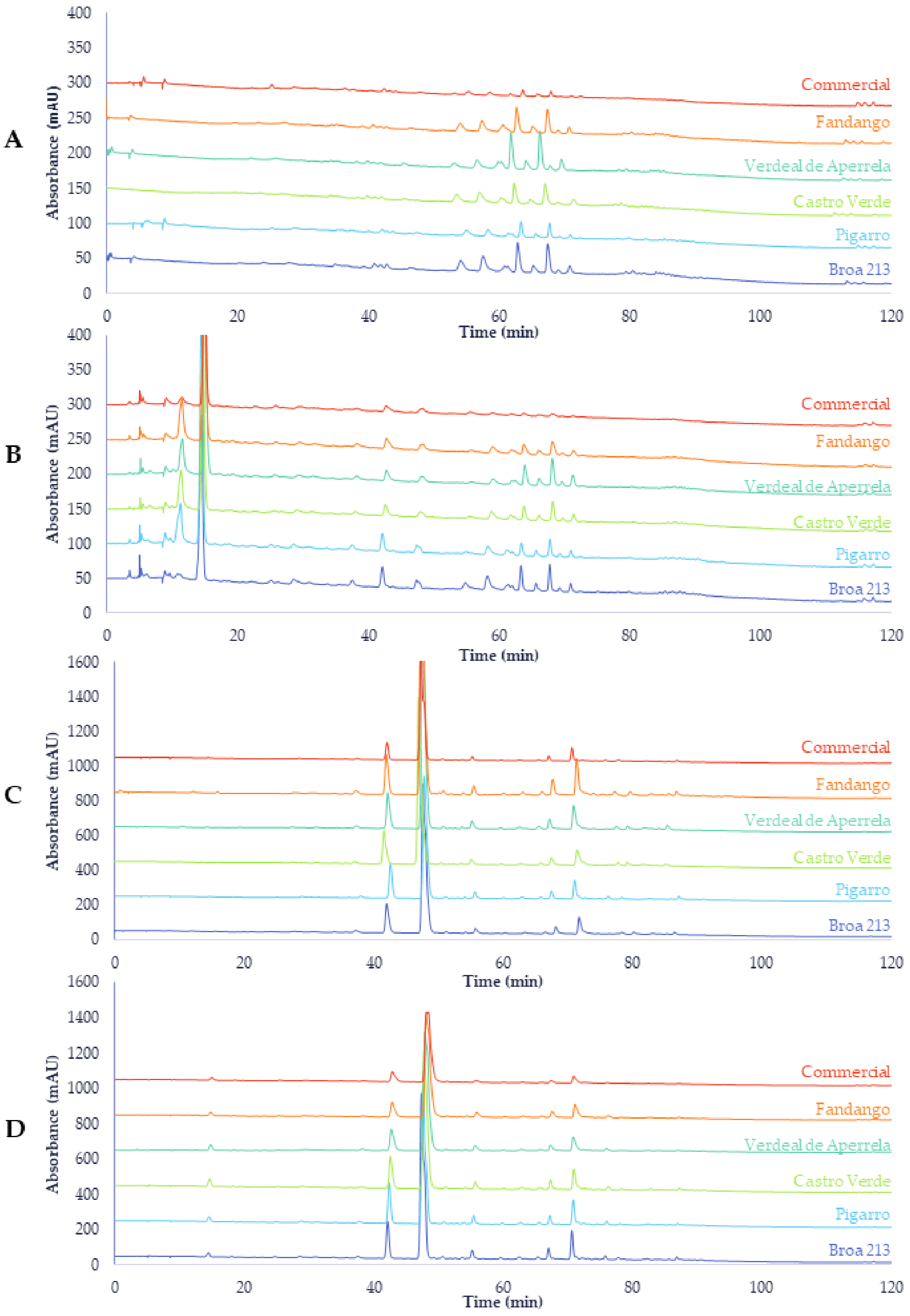

Figure S1. Comparison of chromatographic profiles of (**A**) maize flours soluble fraction (SF), (**B**) *broas* SF, (**C**) maize flour insoluble fraction (IF) and (**D**) *broas* IF at 280 nm.

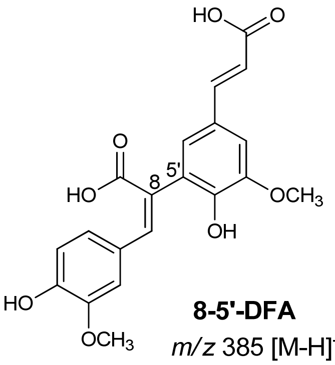

8-5'-DFA
m/z 385 [M-H]$^-$

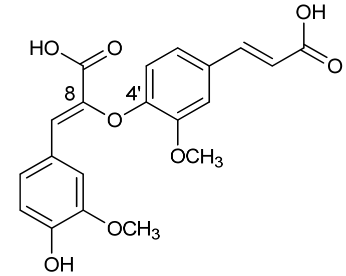

8-O-4'-DFA
m/z 385 [M-H]$^-$

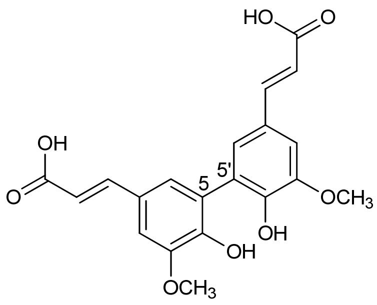

5-5'-DFA
m/z 385 [M-H]$^-$

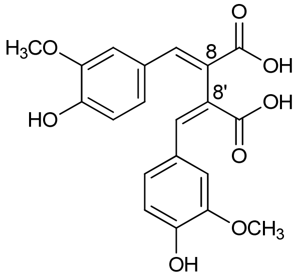

8-8'-DFA
m/z 385 [M-H]$^-$

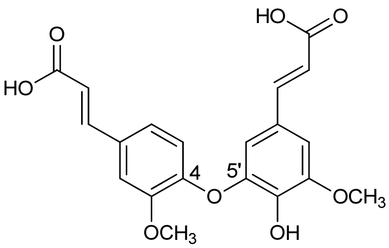

4-O-5'-DFA
m/z 385 [M-H]$^-$

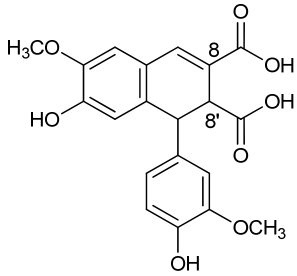

8-8'-DFA$_c$
m/z 385 [M-H]$^-$

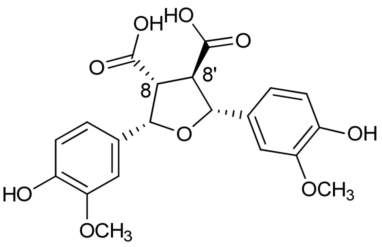

8-8'-DFA$_f$
m/z 403 [M-H]$^-$

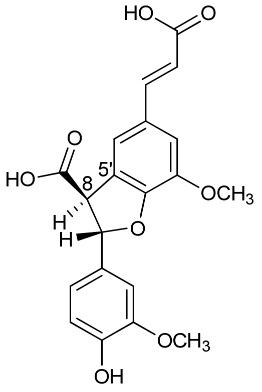

8-5'-DFA$_f$
m/z 385 [M-H]$^-$

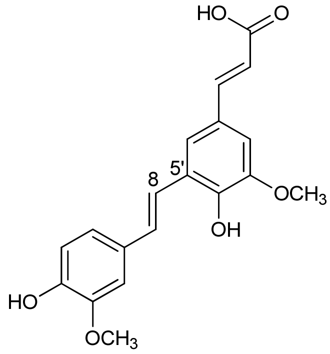

8-5'-DFA$_{dc}$
m/z 341 [M-H]$^-$

Figure S2. Chemical structures of the most known dehydrodiferulic acids (DFAs).

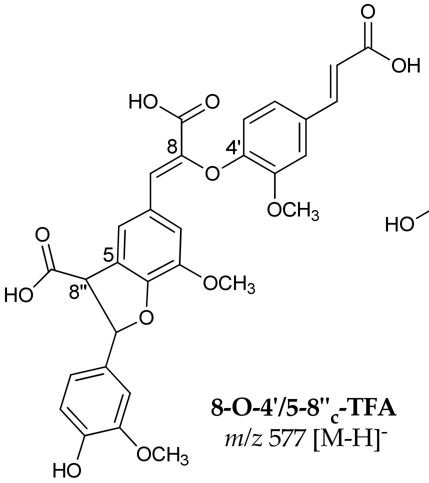

8-O-4'/5-8''$_{c}$-TFA

m/z 577 [M-H]$^{-}$

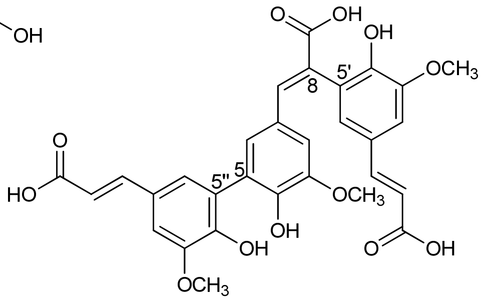

8-5'/5-5''-TFA

m/z 577 [M-H]$^{-}$

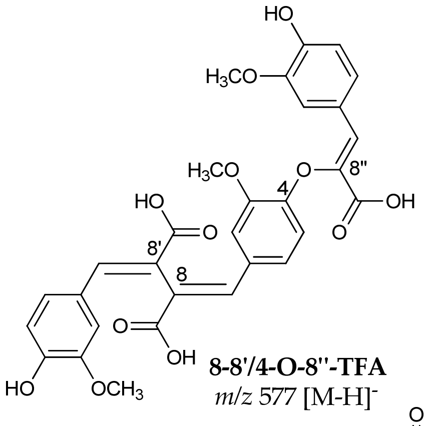

8-8'/4-O-8''-TFA

m/z 577 [M-H]$^{-}$

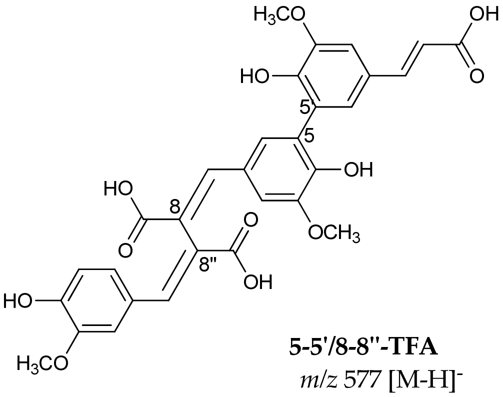

5-5'/8-8''-TFA

m/z 577 [M-H]$^{-}$

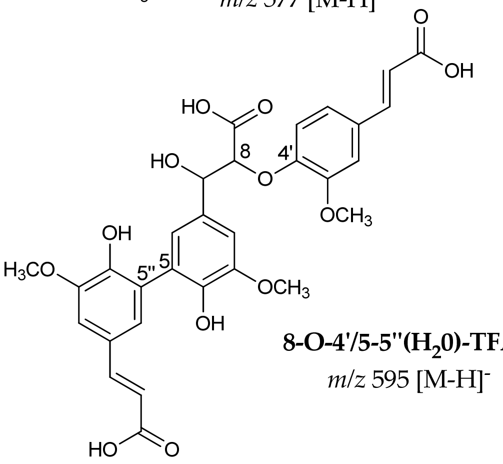

8-O-4'/5-5''($H_{2}0$)-TFA

m/z 595 [M-H]$^{-}$

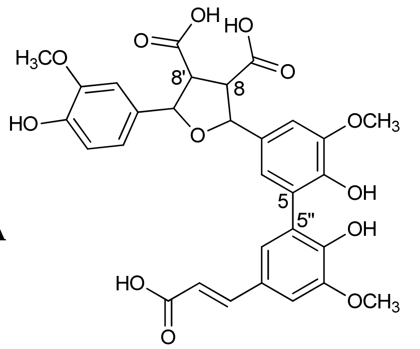

8-8'$_{f}$/5-5''-TFA

m/z 595 [M-H]$^{-}$

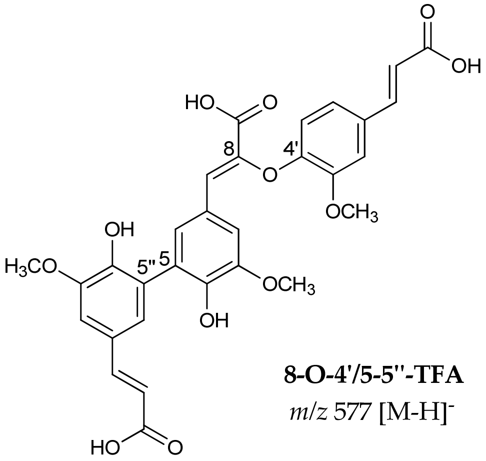

8-O-4'/5-5''-TFA
m/z 577 [M-H]$^-$

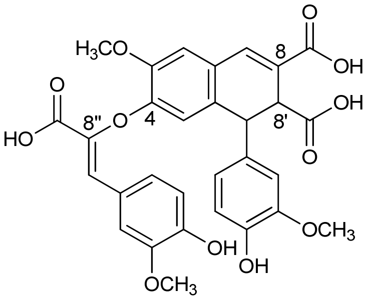

8-8'c/4-O-8''-TFA
m/z 577 [M-H]$^-$

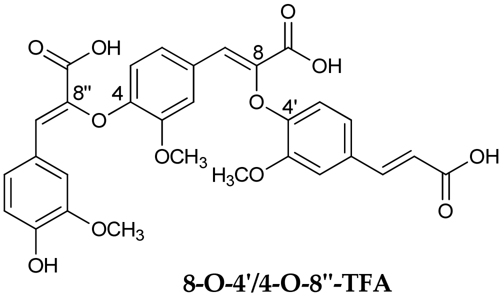

8-O-4'/4-O-8''-TFA
m/z 577 [M-H]$^-$

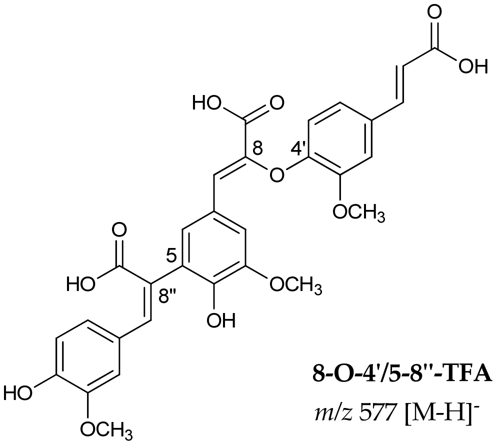

8-O-4'/5-8''-TFA
m/z 577 [M-H]$^-$

Figure S3. Chemical structures of the most known dehydrotriferulic acids (TFAs).

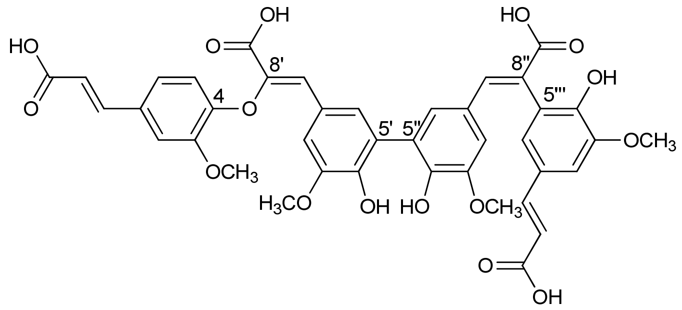

4-O-8'/5'-5''/8''-5'''-TeFA

m/*z* 769 [M-H]$^-$

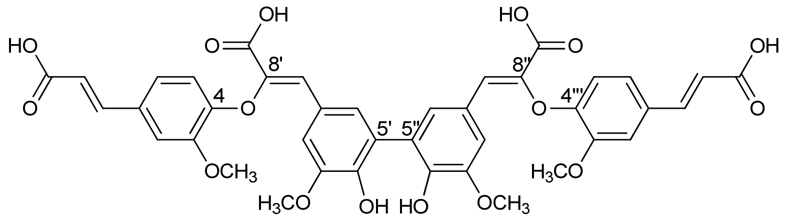

4-O-8'/5'-5''/8''-O-4'''-TeFA

m/*z* 769 [M-H]$^-$

Figure S4. Chemical structures of the most known dehydrotetraferulic acids (TeFAs).

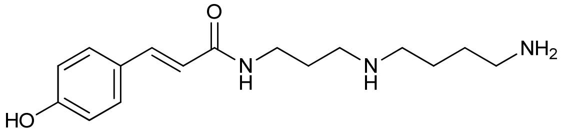

***N*-Coumaroyl spermidine**

m/z 292 [M+H]$^+$

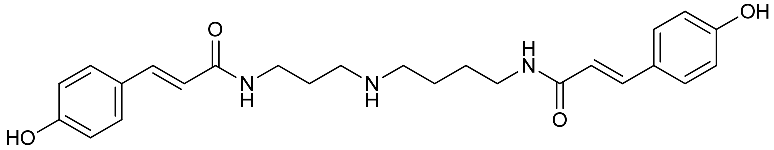

***N,N′*-Dicoumaroyl spermidine**

m/z 438 [M+H]$^+$

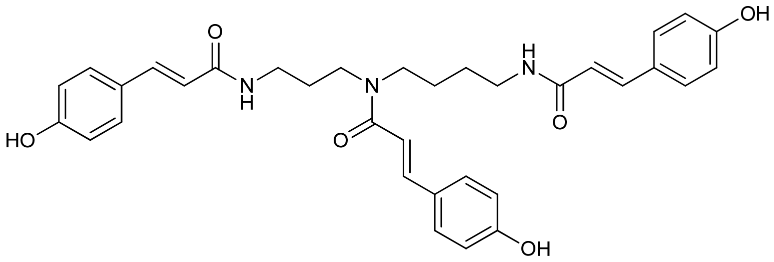

***N,N′*-Tricoumaroyl spermidine**

m/z 584 [M+H]$^+$

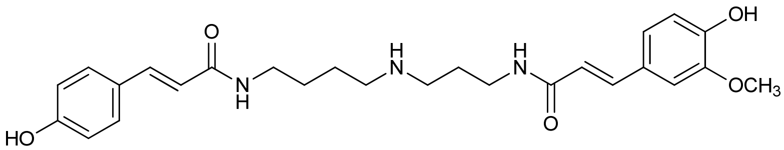

***N,N′*-Coumaroyl feruloyl spermidine**

m/z 468 [M+H]$^+$

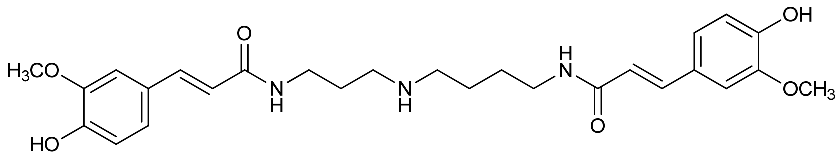

***N,N′*-Diferuloyl spermidine**

m/z 498 [M+H]$^+$

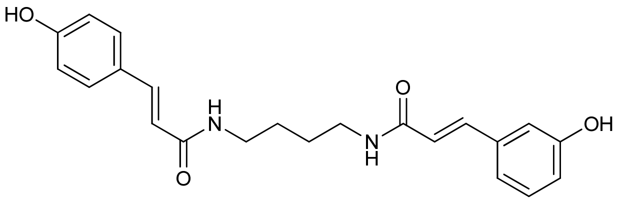

***N,N′*-Dicoumaroyl putrescine**

m/*z* 381 $[M+H]^+$

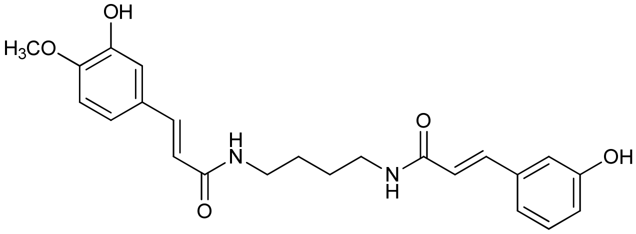

***N,N′*-Coumaroyl feruloyl putrescine**

m/*z* 411 $[M+H]^+$

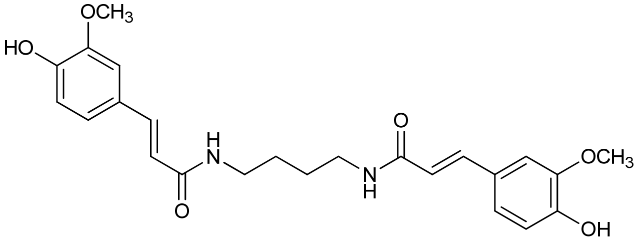

***N,N′*-Diferuloyl putrescine**

m/*z* 441 $[M+H]^+$

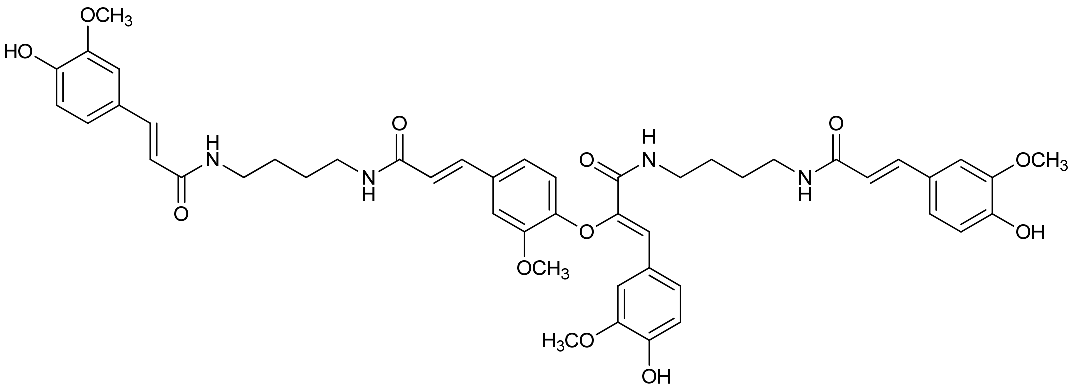

***bis-N,N′*-Diferuloyl putrescine**

m/*z* 877 $[M-H]^-$

Figure S5. Molecular structures of soluble hydroxycinnamic acid amides (HCAAs).

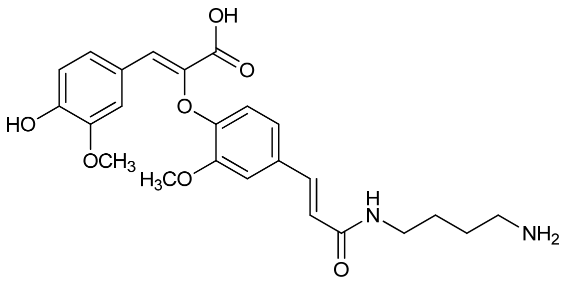

***N*-8-O-4'-Dehydrodiferuloyl putrescine**

m/z 457 $[M+H]^+$

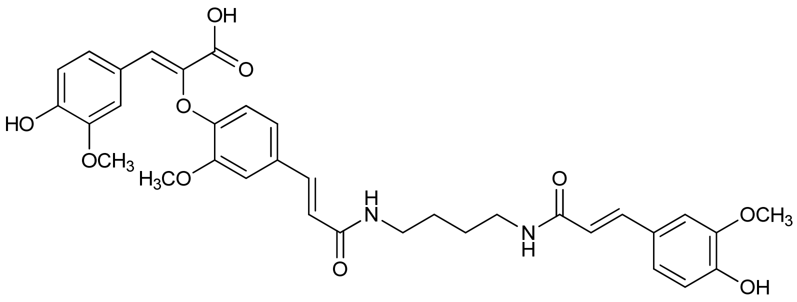

***N,N′*-Feruloyl 8-O-4'-didehydrodiferuloyl putrescine**

m/z 633 $[M+H]^+$

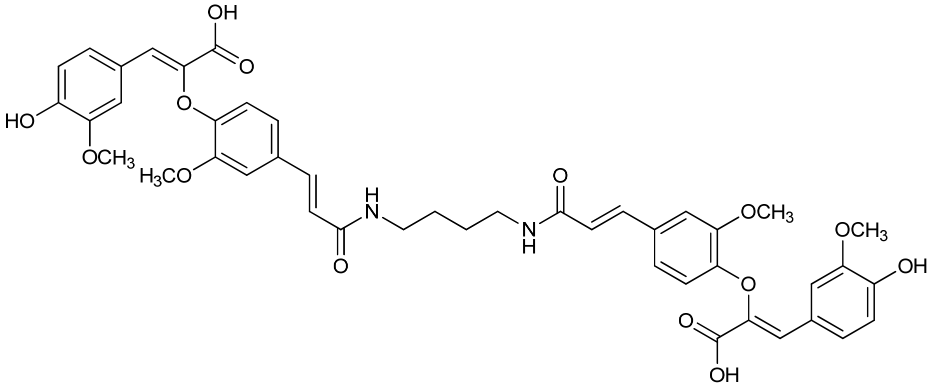

***N,N′*-8-O-4'-Didehydrodiferuloyl putrescine**

m/z 825 $[M+H]^+$

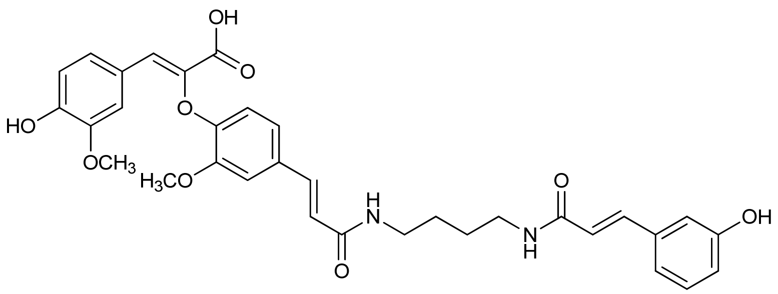

***N,N′*-Coumaroyl 8-O-4'-dehydrodiferuloyl putrescine**

m/z 603 $[M+H]^+$

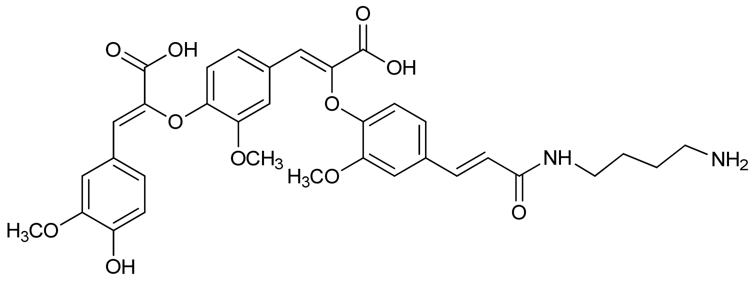

***N*-8-O-4'/4-O-8''-Dehydrotriferuloyl putrescine**

m/z 649 $[M+H]^+$

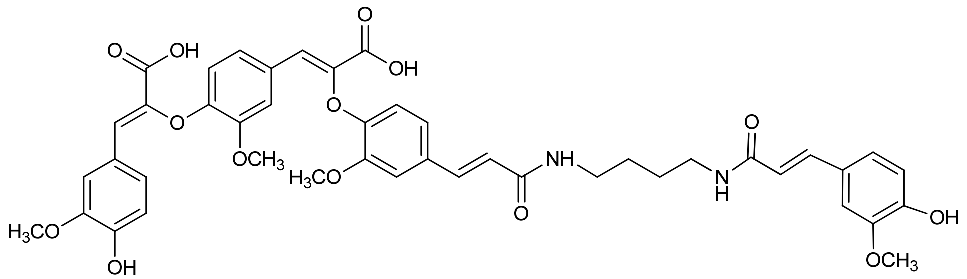

***N,N′*-Feruloyl 8-O-4'/4-O-8''-dehydrotriferuloyl putrescine**

m/z 825 $[M+H]^+$

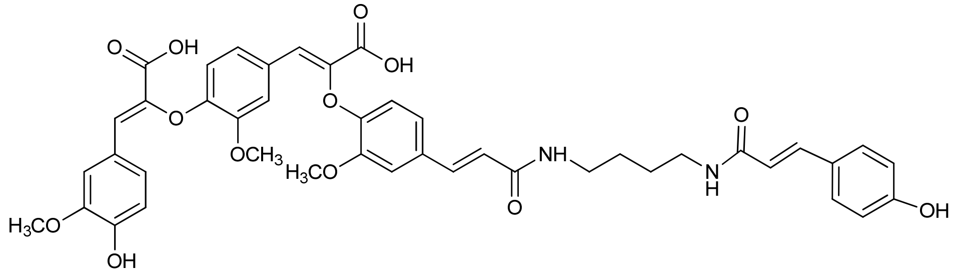

***N,N′*-Coumaroyl 8-O-4'/4-O-8''-dehydrotriferuloyl putrescine**

m/z 795 $[M+H]^+$

Figure S6. Chemical structures suggested for 8-O-4′-dehydrodiferulic and 8-O-4′/4-O-8′′-dehydrotriferulic acid putrescines.

Table S1. Details of the MRM conditions applied in the HPLC-DAD-MS/MS analysis.

Compound	*m/z*	MS/MS ions	Cone Voltage (V)	Collision Energy (eV)
Ferulic acid (−)	193	134	30	10
p-Coumaric acid (−)	163	93	20	20
o-Coumaric acid (−)	163	93	20	20
m-Coumaric acid (−)	163	93	20	20
p-Hydroxybenzoic acid (−)	137	93	30	10
Caffeic acid (−)	179	135	20	10
Syringic acid (+)	199	140	20	10
Vanillic acid (+)	169	93	20	10
Protocatechuic acid (−)	153	109	20	10
Citric acid (−)	191	111	10	10
Gallic acid (−)	169	125	20	10
Syringaldehyde (+)	183	123	20	10
Vanillin (+)	153	93	20	10
Quercetin (−)	301	151	30	20
Kaempferol (−)	285	178	40	30
Citric acid (−)	191	111	10	10

(+) Compounds analysed in positive ion mode; (−) Compounds analysed in negative ion mode.
